# A Triboelectricity-Driven Self-Sustainable System for Removing Heavy Metal from Water

**DOI:** 10.3390/mi17020229

**Published:** 2026-02-11

**Authors:** Jonghyeon Yun, Hyunwoo Cho, Geunchul Kim, Inkyum Kim, Daewon Kim

**Affiliations:** 1 Department of Electronics and Information Convergence Engineering, Kyung Hee University, 1732 Deogyeong-daero, Giheung-gu, Yongin 17104, Republic of Korea; 2 Department of Semiconductor Engineering, Kyung Hee University, 1732 Deogyeong-daero, Giheung-gu, Yongin 17104, Republic of Korea; 3 Department of Electronic Engineering, Institute for Wearable Convergence Electronics, Kyung Hee University, 1732 Deogyeong-daero, Giheung-gu, Yongin 17104, Republic of Korea

**Keywords:** triboelectric nanogenerator, liquid–solid, water purification, electrochemical deposition, self-powered

## Abstract

As the demand for clean water grows, the strategic management of water resources has become increasingly critical. However, the depletion of these resources is being accelerated by anthropogenic pollutants and resultant internal pipe corrosion within distribution networks. Conventional water treatment methods are characterized by high energy consumption, rendering them impractical in environments lacking a continuous external power supply. Consequently, innovative, self-sustained technologies for simultaneously monitoring fluid conditions and purifying water are a necessity. In this work, we present a water-driven triboelectric nanogenerator (W-TENG) used for energy harvesting and water-quality monitoring within pipe networks. Composed of a silicone rubber tube and aluminum electrodes, the optimized W-TENG achieved an open-circuit voltage of 58 V, short-circuit current of 1.1 µA, and 59.5 mW/m^2^ at a 10 MΩ load. The W-TENG distinguishes pH levels and liquid types based on electrical outputs. Notably, a parallel connection of two W-TENGs enhanced electrical energy by 214% compared to the sum of two units. As an application, a self-powered electrochemical deposition was conducted and copper ions were successfully removed using energy stored in a 1 mF capacitor. These results indicate that the W-TENG is expected to be utilized as a self-powered platform for simultaneous water purification and real-time infrastructure monitoring.

## 1. Introduction

Clean water is an indispensable resource for human survival and industrial sectors, and its strategic management has become increasingly critical agenda intensifying global water scarcity. In modern infrastructure, these water resources are primarily distributed through extensive pipe networks. However, these vital resources are being rapidly depleted and contaminated as they travel through the distribution system. Specifically, water quality is significantly deteriorated by diverse anthropogenic pollutants flowing from urban environments, including domestic sewage, livestock waste, industrial effluents, and automotive emissions, which introduce organic pollutants and heavy metals into the supply. As these contaminants are transported without adequate treatment, they accelerate the structural degradation of the infrastructure, e.g., internal pipe corrosion. This degradation not only introduces secondary pollutants but also leads to significant water loss through leakage, further accelerating the depletion of available resources. Despite advancements in conventional water treatment, high operational costs and heavy reliance on external power sources limit their deployment, particularly in underdeveloped regions. Conventional water treatment relies on a multi-stage framework comprising physical filtration, chemical coagulation, and biological degradation [1,2]. While effective at a large scale, these centralized systems are inherently energy-intensive, requiring substantial external power for high-pressure membrane operation and ultraviolet sterilization. Furthermore, the extensive use of chemical reagents often leads to the formation of secondary pollutants, such as toxic sludge and disinfection by-products, which necessitate additional complex management. These traditional methods are designed for centralized facilities, leaving them incapable of addressing real-time contamination or structural degradation that occurs during transit through distribution pipe networks. Additionally, pollutants such as heavy metals are difficult to treat using conventional methods. Hence, new technologies for water purification are required, reducing the energy consumption issues as well as monitoring water quality in the pipe.

Recently, energy-harvesting technologies have emerged as a suitable solution to scavenge the dissipated energy that surrounds us. Extensive research has been conducted to scavenge various energy sources based on photovoltaic [3,4,5,6], thermoelectric [7,8,9,10,11,12], piezoelectric [13,14,15,16,17,18], pyroelectric [19,20,21,22], and triboelectric effects [23,24,25,26,27,28,29,30,31,32]. In water-transported infrastructure, vast quantities of water are transported through extensive piping networks; however, the associated water-kinetic energy is constantly dissipated without proper utilization. Therefore, by using energy-harvesting technologies, the energy losses inherent in piping networks can be utilized for energy demands. Among various energy-harvesting technologies, the triboelectric nanogenerator (TENG) can be considered as a suitable harvester to scavenge dissipated water-kinetic energy. The working mechanism of a TENG relies on the coupling of contact electrification and electrostatic induction, triggered by the mechanical interaction between two materials with distinct electron affinities [33,34]. This simple operating principle allows for the utilization of a wide array of materials and facilitates cost-effective, facile fabrication, providing an advantage in harvesting mechanical energy with low frequency, such as irregular fluidics. Owing to the great potential of TENGs, various liquid–solid TENGs have been investigated [35,36,37,38,39,40,41,42,43]. Generally, these liquid–solid TENGs use flowing water to move a structure such as a fan, or electricity is generated as the liquid–solid TENG moves through water. However, they are difficult to integrate into conventional water facilities such as piping systems due to their complex structures. Fan structures hinder the water flow in the piping system and floating structures are difficult to insert into the pipe [40]. Hence, a liquid–solid TENG is required to harvest the dissipated energy at the piping networks.

Liquid–solid TENGs integrated with pipe frameworks have been extensively explored for infrastructure monitoring. Previous research has primarily focused on non-invasive liquid–solid TENG configurations in order to detect internal phenomena such as bubble formation through a simplified architecture [38,39]. However, these non-invasive designs are inherently limited in their ability to analyze the chemical properties or corrosive potential of the internal fluid. Existing invasive models often operate on a closed-cycle basis, rendering them unsuitable for continuous-flow piping systems [41]. Therefore, there is a critical need for a novel liquid–solid TENG that synergizes the advantages of both approaches, enabling sustainable energy harvesting from continuous flow while simultaneously providing real-time monitoring of fluid conditions within the pipe.

Herein, a water-driven TENG (W-TENG) was demonstrated with a facile fabrication process. The fabricated W-TENG generated an open-circuit voltage (*V*_OC_) of 58 V and a short-circuit current (*I*_SC_) of 1.1 µA and 59.5 mW/m^2^ at a load resistance of 10 MΩ with the flowing water. Also, the structure of the W-TENG was optimized by adjusting the distance between two electrodes, the length of the electrode, and the water flow rate. The electrical outputs were investigated and analyzed according to the pH concentration, and the W-TENG was used to successfully distinguish various liquids based on the electrical output. The electrical outputs with series and parallel connections of the W-TENGs were investigated. Notably, the electrical energy in the capacitor showed a 214% increase compared to the sum of electrical energy of two-unit W-TENGs. Using the great potential of W-TENG as an energy source, the electrochemical deposition was conducted to remove the heavy metal in the water. The W-TENG charged a 1 mF capacitor to 4.5 V for 4 h, and heavy metal ions (Cu ions) were successfully removed from the water using electrical energy at the 1 mF capacitor. Considering these results, the fabricated W-TENG is expected to be utilized for self-powered water purification and for monitoring water status in pipe networks based on its great potential for monitoring and its self-powered electrochemical deposition.

## 2. Materials and Methods

A commercial silicone–rubber tube with a diameter of 8 mm was utilized to imitate the pipe in the piping networks. Based on this silicone–rubber tube, an aluminum (Al) electrode was warped to form a source electrode. Then, the drain electrode with a pointed end penetrated the silicone rubber tube at a certain distance from the source electrode. As a result, the W-TENG was fabricated with a facile fabrication process. To imitate pollutants, urea (Sigma-Aldrich, St. Louis, MO, USA) and ethanol (SAMCHUN, Pyeongtaek, Gyeonggi-do, Republic of Korea) were used. To conduct self-powered electrochemical deposition, sulfuric acid (Duksan Pure Chemicals, Jincheon, Chungcheongbuk-do, Republic of Korea) and copper sources (VTM, Incheon, Republic of Korea) were utilized. To confirm the pH concentration, a pH meter (STARTER 3100, OHAUS, Parsippany, NJ, USA) was employed. A scanning electron microscope (MERLIN, Carl Zeiss, Jena, Germany) was used to observe the Cu-deposited surface of the Al electrode. The electrical output was measured using an electrometer (Keithley Model 6514, Cleveland, OH, USA).

## 3. Results and Discussion

**Figure 1:**
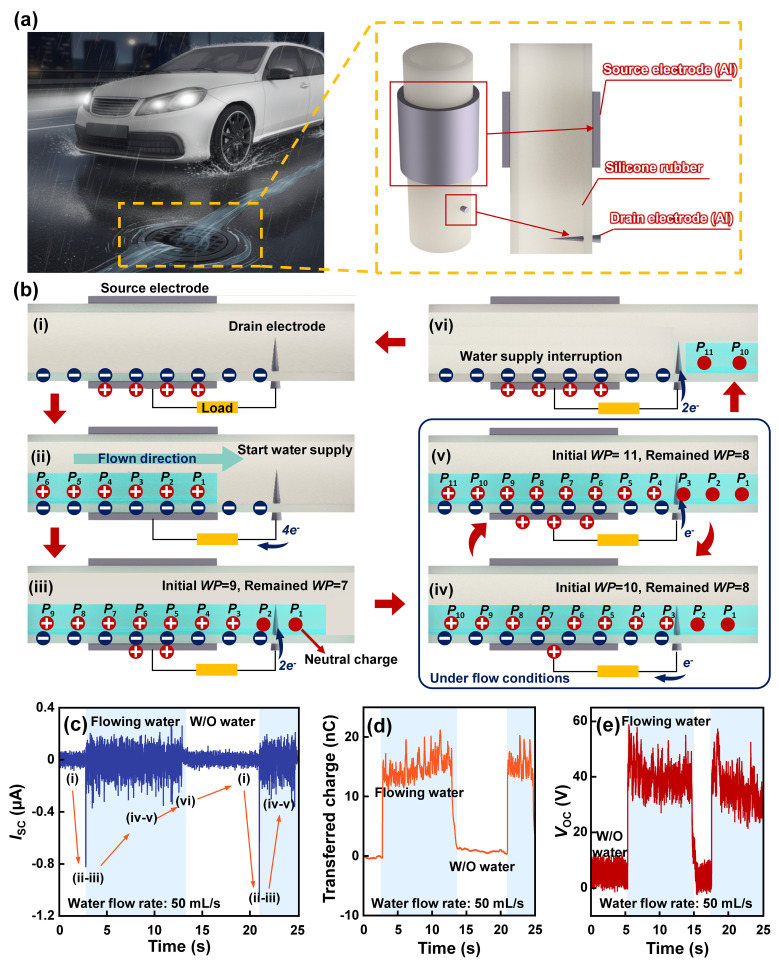
(**a**) Illustration of a W-TENG, including its components and its applicable environment. (**b**) Working mechanism of the proposed W-TENG according to the water supply. *WP* represents positive charges in the water and *P*_N_ indicates the order of positive charges. The (**c**) *I*_SC_, (**d**) *Q*_SC_, and (**e**) *V*_OC_ are generated from the W-TENG. The blue region indicates the area with flowing water and the white region indicates the absence of water in the pipe.

Figure 1a shows an illustration of water flowing into a storm drain in a city. This flowing water will continuously flow into the drainpipe, providing ambient energy. At the drainpipe, a water-driven triboelectric nanogenerator (W-TENG) can be installed to scavenge this energy. The W-TENG was composed of a source electrode, a silicon rubber tube, and a drain electrode, respectively. The thin and sharp shape of the drain electrode is designed to not disturb the flow of water in the pipe networks. Figure 1b illustrates the operational mechanism of the W-TENG in converting water flow kinetic energy into electrical power. As illustrated in Figure 1b, the process transitions through several distinct phases: (i) no water is initially supplied; (ii–iii) flow is initiated; (iv–v) electricity is consistently generated under a stable flow regime; and (vi) water is drained once the supply is terminated. In the initial state (Figure 1b(i)), the silicone rubber is negatively charged, while the source electrode accumulates positive charges to maintain electrostatic equilibrium. Upon making contact with the silicone rubber (Figure 1b(ii)), the water acquires positive charges, denoted as *P*_N_ (where *N* is a natural number), through triboelectric interaction. To compensate for this potential shift, electrons are pumped from the drain electrode to the source electrode. As the water subsequently contacts the drain electrode (Figure 1b(iii)), electrons are driven from the source to the drain to neutralize the positive charges in the water, specifically *P*_1_ and *P*_2_. Despite the continuous flow, a finite time is required for the charge transfer between the water and the other surfaces, inducing a transient electrical imbalance. For instance, the generation of an additional positive charge (*P*_10_) in Figure 1b(iv) triggers a subsequent electron migration from the drain back to the source. This dynamic process continues as shown in Figure 1b(v), where the charging of *P*_11_ is offset by the neutralization of *P*_3_, thereby stabilizing the total charge count in Figure 1b(iv). Finally, as the water flow ceases, the charge distribution on the source electrode returns to its original state to restore the final electrical equilibrium, as shown in Figure 1b(vi). Figure 1c–e demonstrate the proposed working mechanism of the W-TENG. In particular, the short-circuit current (*I*_SC_) was generated by following each state of the proposed working mechanism as water flowed. As a result, the W-TENG generated an *I*_SC_ of 1.1 µA and an open-circuit voltage (*V*_OC_) of 58 V with a water flow rate of 50 mL/s. The experimental configuration of W-TENG is provided in Figure S1. Considering these results, the proposed W-TENG can generate electricity from continuously flowing water.

**Figure 2:**
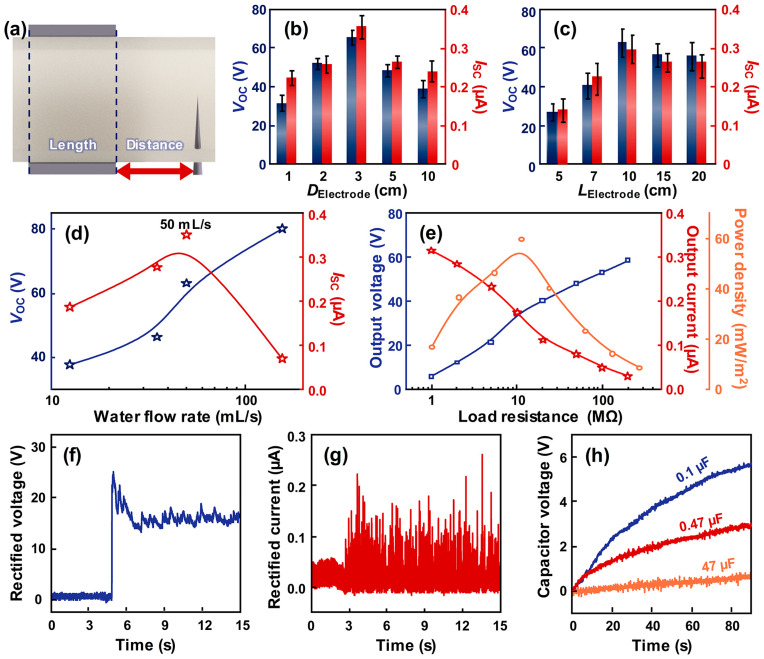
(**a**) Inner structure of the W-TENG and its adjustable factors. (**b**) Measured electrical outputs generated from the W-TENG by varying distance between source electrode and drain electrode. (**c**) Measured electrical outputs generated from the W-TENG according to the length of the source electrode. (**d**) Measured electrical outputs of the W-TENG with various water flow rates. (**e**) Electrical power density of the W-TENG according to the load resistance. Rectified (**f**) voltage and (**g**) current of the W-TENG. (**h**) Comparison of capacitor voltages charged at various commercial capacitors using the W-TENG.

To increase the electrical output generated by the W-TENG, the structure of the W-TENG should be optimized. In Figure 2, the W-TENG is optimized to enhance the electrical output. Due to its simple structure, the optimization of the W-TENG can be conducted by varying the length of the source electrode (*L*_Electrode_) and the distance between the source and drain electrode (*D*_Electrode_), as shown in Figure 2a. These two parameters are considered based on the working mechanism of the W-TENG. The *V*_OC_, *I*_SC_, and transferred charge in the short-circuit state (*Q*_SC_) generated from the TENG can be theoretically calculated with the formula shown below [44]:

(1) $$V_{\mathrm{OC}}=\frac{\sigma S}{2C}$$

(2) $$I_{\mathrm{SC}}=\frac{dQ_{\mathrm{SC}}}{dt}$$

(3) $$Q_{\mathrm{SC}}=\frac{\sigma S}{2}$$

where the *σ*, *S*, and *C* correspond to surface charge density, contact area, and capacitance, respectively. The *D*_Electrode_ can affect the electrical output of W-TENG because as the water flows, the water on the surface mixes due to convection, etc., which can cause changes in the surface charge density. The electrical output generated from the W-TENG can also be affected by the *L*_Electrode_ because the surface charge density of the water can be varied. In Figure 2b, the electrical outputs generated from the W-TNEG according to the *D*_Electrode_ are provided. From 1 cm to 3 cm of *D*_Electrode_, the electrical output showed an increased trend. This is because of the short distance required to achieve a sufficient triboelectric effect between the water and silicon rubber. As shown in Figure 1b, water becomes positively charged at the interface with the silicone rubber. Hence, with the short *D*_Electrode_, the amount of positive charge inevitably reduces, inducing decreased electrical output. When the *D*_Electrode_ is increased above 3 cm, the positive charges of the water are reduced due to the charge recombination in the water. Because flowing water possesses a dynamic surface, circulation occurs within the water as it flows. During this circulation, the positive charges combine with the negative ions in the water, neutralizing them and reducing electrical output. Also, in contrast to solids, liquid materials possess a dynamical contact surface. The passage of water through the source electrode facilitates contact with the silicone rubber, thereby inducing additional positive charge accumulation at the water interface and enhancing the overall charge density. Due to this sufficient triboelectric effect, the electrical output increased up to a *D*_Electrode_ of 3 cm. When the *D*_Electrode_ is continually increased, the contact area between water and W-TENG is also increased. However, due to the fluidity of water, the new water interface is introduced and is positively charged by new triboelectrification. However, in the water, there were previously formed positive charges, inducing the screening effect. As a result, although the overall electrical neutrality is maintained, the effective surface charge density is reduced due to the screening effect, which reduces the electrical output of the TENG generated by charge transfer on the surface. Hence, the 3 cm distance of *D*_Electrode_ is optimized to generate the highest electrical output. In Figure 2c, the effect of *L*_Electrode_ into the electrical output is investigated. From 5 cm to 10 cm, the electrical output generated from the W-TENG increased. This increase in electrical output is attributed to the increased surface charges resulting from an enlarged contact area. However, when *L*_Electrode_ exceeds 10 cm, the effective surface contact area begins to decrease, thereby reducing the effective surface charge density. Theoretically, the electrical output of a TENG is expected to scale proportionally with the contact area. However, interfacial irregularities and screening effects inherent to the fluidic properties of water can lead to a reduction in surface charge density as the area expands. Consequently, for *L*_Electrode_ exceeding 10 cm, the gain from the increased contact area is offset by the diminished charge density, leading to a saturation of the electrical output. Based on these findings, the W-TENG structure was optimized with a *D*_Electrode_ of 3 cm and an *L*_Electrode_ of 10 cm.

In terms of electrical output, the input source also should be considered. In Figure 2d, the electrical outputs according to the water flow rate are investigated. As the water flow rate increases, the electrical output generated from W-TENG increases, and the highest electrical output is observed at a water flow rate of 50 mL/s. The presence of an air gap within the pipe significantly influences the electrical output by providing sufficient volume for hydrodynamic circulation. When an air gap exists, the water initially positively charged via friction with the silicone rubber can undergo convective mixing and recirculate away from the interface. This dynamic movement facilitates continuous charge separation and induces further charge transfer at the surface, maintaining a steady current flow. In contrast, when the pipe is completely filled, the lack of void space constrains the movement of water, effectively suppressing internal circulation. In this regime, although a high electrostatic potential is established and maintained at the water–silicone interface, Since the water, which is already charged with positive charges, continues to move, no new charge transfer occurs. Consequently, despite the high potential, the magnitude of the current decreases rapidly. Also, the highest transferred charge was observed at a water flow rate of 50 mL/s, as shown in Figure S2. With the water flow rate of 50 mL/s, electrical power density was measured according to the load resistance, and it generated the highest value of 59.5 mW/m^2^ at a load resistance of 10 MΩ. Also, its rectified voltage and current are investigated in Figure 2f,g. The rectified electrical outputs decreased before rectifying due to losses from the rectifier. The W-TENG generated an electrical output with a higher average value using continuously flowing water. With these advantages, the capacitor-charging ability of W-TENG was investigated to evaluate its potential as an energy source, and it successfully charged the commercial capacitor. These results indicate that the proposed W-TENG can be utilized as the energy source for water purification.

**Figure 3:**
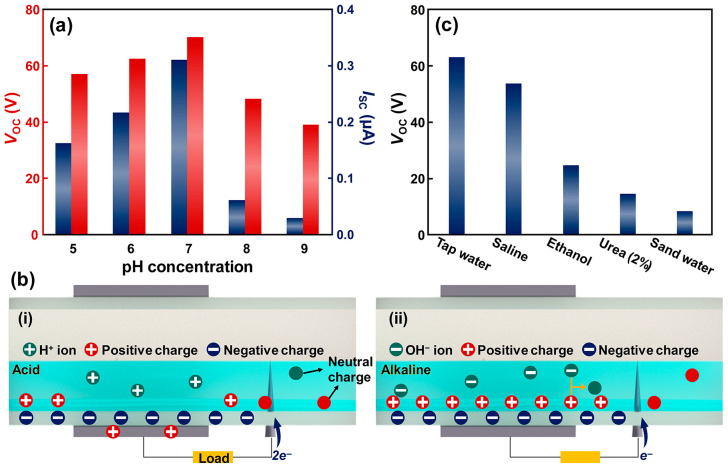
(**a**) Electrical outputs according to the pH concentration. (**b**) Illustration to describe the internal situation in the W-TENG with (**i**) acid and (**ii**) alkaline solutions, respectively. (**c**) Electrical outputs with various liquids.

Monitoring liquid conditions within piping networks is essential for maintaining water quality and preventing infrastructure corrosion. Among various indicators, the pH level of water significantly influences the degree of pipe degradation. As illustrated in Figure 3a, the electrical outputs of the W-TENG were measured according to the pH concentration. The highest output was observed in a neutral state (pH 7), where the screening effect from free ions was minimal. As the solution became increasingly acidic or alkaline, the electrical output decreased; however, the W-TENG with acidic solutions generated higher outputs compared to alkaline solutions. This result is attributed to the distinct screening behaviors of hydrogen (H^+^) and hydroxide (OH^−^) ions. According to the operating mechanism of the W-TENG, water becomes positively charged upon contact with the silicone rubber tube. In alkaline solutions, the negatively charged OH^−^ ions possess electrical properties opposite to the charged water, thereby hindering the formation of a potential difference more severely than H^+^ ions in acidic solutions. Consequently, the presence of OH^−^ ions in alkaline media leads to a more pronounced reduction in electrical output (Figure 3b). Furthermore, while H^+^ ions also interfere with potential difference formation, they can contribute to current generation by donating electrons to the drain electrode, resulting in relatively higher outputs than in alkaline environments. The electrical outputs were also investigated for various liquid types (Figure 3c). Tap water exhibited the highest output, primarily due to its high dielectric constant compared to ethanol, which facilitates stronger electrostatic induction. Common contaminants such as urea and sand-dispersed water were also tested. Both urea and sand water induced a screening effect that reduced the effective surface charge density at the liquid–solid interface, leading to diminished electrical outputs. These findings demonstrate that the proposed W-TENG is a capable platform for real-time monitoring of liquid states and contamination levels within pipe networks.

**Figure 4:**
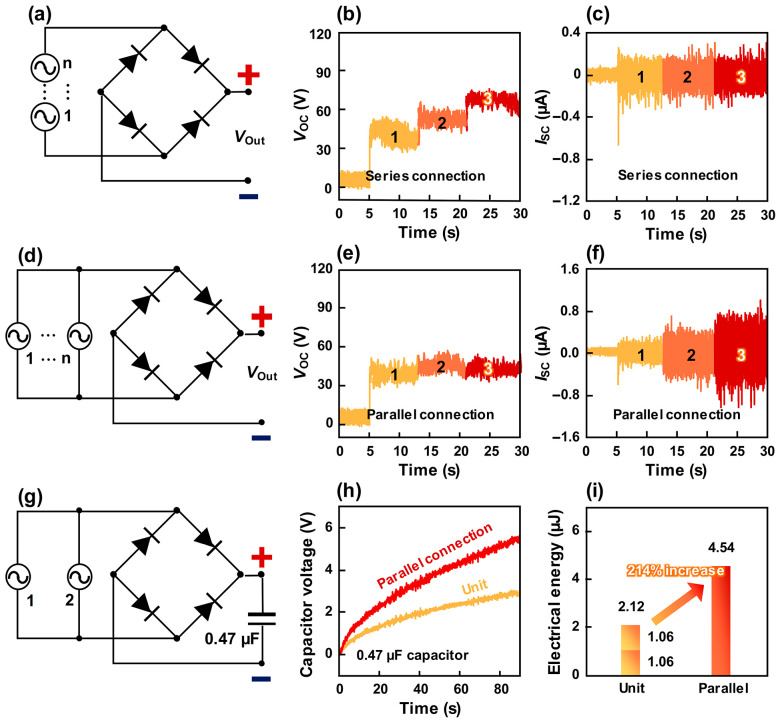
(**a**) Circuit diagram of the multiple W-TENGs in series connection. (**b**) *V*_OC_ and (**c**) *I*_SC_ acquired by sequentially connecting three W-TENGs in series. (**d**) Circuit diagram of the multiple W-TENGs in parallel connection. (**e**) *V*_OC_ and (**f**) *I*_SC_ acquired by sequentially connecting three W-TENGs in parallel. (**g**) Circuit diagram of parallel connection of W-TENG. (**h**) Comparison of capacitor voltages charged by two W-TENGs with parallel connection and single W-TENG, and (**i**) stored electrical energy. For comparison, the electrical energy stored by a single W-TENG was doubled.

Despite the high electrical performance of a single W-TENG, enhancing the output through systematic configuration is essential for practical applications. Figure 4 illustrates the scalability of the W-TENG system using multiple units connected in series and its outputs (Figure 4a–c) or in parallel with its outputs (Figure 4d–f). In a series configuration of three units, the *V*_OC_ increased linearly while the *I*_SC_ remained constant, as shown in Figure 4b,c. Conversely, in a parallel configuration, the *I*_SC_ scaled up while the *V*_OC_ remained stable (Figure 4e,f). Given that TENGs inherently generate high voltage but low current, we utilized a parallel connection of two W-TENGs to compensate for the current limitations when charging a 0.47 µF capacitor (Figure 4g). After 60 s of charging, the parallel-connected units reached 4.4 V, significantly outperforming the 2.12 V achieved by a single unit, as shown in Figure 4h. The stored electrical energy (*E*_Cap_) in the capacitor was calculated using the following equation:

(4) $$ECap=\frac{1}{2}CV^{2}$$

where *C* and *V* are capacitance and capacitor voltage. The parallel configuration generated a stored electrical energy of 4.54 µJ, whereas a single W-TENG generated only 1.06 µJ. Notably, the energy from the parallel system represents a 214% increase compared to the theoretical sum of two individual units (2.12 µJ), as shown in Figure 4i. This synergistic enhancement in energy storage efficiency, rather than a mere linear summation, highlights the excellent potential of W-TENG systems as a scalable and robust energy source for practical, self-sustainable applications.

**Figure 5:**
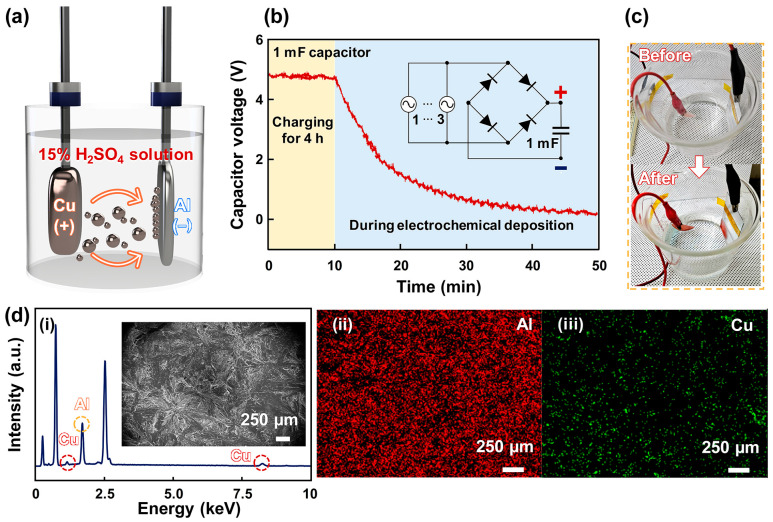
(**a**) Illustration of the self-powered electrochemical deposition using the W-TENG. (**b**) Measured capacitor voltage before and during self-powered electrochemical deposition. The inset shows the circuit diagram for charging capacitor. (**c**) Result of self-powered electrochemical deposition. (**d**) EDX analysis results: (**i**) an EDX layered electron image including SEM images (inset) and elemental mapping for (**ii**) Al and (**iii**) Cu.

To evaluate the practical viability of the proposed W-TENG in real-world applications, its long-term durability was tested under a continuous-flow environment for 4 h as shown in Figure S3. The results showed no significant degradation in electrical output, demonstrating its robust performance in continuous-flow conditions. Consequently, these findings confirm that the proposed W-TENG is capable of consistent energy-harvesting in both fluctuating and continuous-flow conditions. Then, a self-sustainable electrochemical deposition was demonstrated using the W-TENG, as shown in Figure 5a. Exhaust fumes from cars include heavy metals, which accumulate on roads and run off when it rains, entering pipe systems and polluting the water. To mimic this, the Cu source was ionized in 15% sulfuric acid solution [45]. Then, these ionized Cu ions were removed through self-powered electrochemical deposition using the electrical energy produced by the W-TENG. To conduct the self-powered electrochemical deposition, a 1 mF capacitor was charged using the W-TENG for 4 h, and it reached 4.5 V, as shown in Figure 5b. The electrochemical deposition was conducted for approximately 40 min. As a result, Cu ions were deposited on the Al electrode, as shown in Figure 5c. To evaluate the electrochemical deposition, adsorption capacity (*q*_t_), kinetics (*k*), energy consumption per mass of removed contaminant (*ECM*), and removal efficiency (*E*_R_) were investigated. These values are provided in Table 1. The detailed formulas and definition of each parameter are provided in Figure S4, and Tables S1 and S2. According to Faraday’s law, the required charge and energy can be calculated with the formula shown below:

(5) $$Q=\frac{m}{Z}$$

(6) E=QV

where *Q*, *m*, *Z*, *E*, and *V* represent the charge, the mass of the deposited substance (0.0065 g), the electrochemical equivalent of Cu (0.0003296 g/C), energy, and voltage. Hence, the *Q* is 19.7 C and required energy is 29.5 J when 1.5 V is applied to electrochemical deposition. Notably, although total energy delivered by the W-TENG was numerically smaller than the theoretical value (29.5 J) for standard electrochemical deposition, the Cu layer was deposited on the Al electrode. This result may be due to electric field-accelerated ion migration boosting the galvanic replacement. In accordance with reference [46], where even a low voltage of 0.26 V boosted ion movement, the high instantaneous voltage (4.5 V) provided by the proposed W-TENG created a strong electric field that facilitated the deposition process beyond Faraday’s primary expectations. Despite these interesting findings, this phenomenon will be handled in future work. To confirm the electrochemical deposition, the energy-dispersive X-ray spectroscopy (EDX) was conducted using a scanning electron microscope. Then, the Cu peaks were confirmed on the Al electrode, as shown in Figure 5d. These results demonstrated the potential of the proposed W-TENG, which can remove the heavy metals in water with dissipated water energy.

## 4. Conclusions

In summary, a high-performance water-driven triboelectric nanogenerator (W-TENG) was strategically engineered to harvest water-kinetic energy from fluid flow within municipal or industrial pipe networks. The device architecture employs silicone rubber as the primary negative triboelectric layer, which is integrated with an aluminum-based source and drain electrodes. This design facilitates efficient charge induction and transfer at the liquid–solid interface during continuous water flow. The fundamental working mechanism of the W-TENG was systematically validated through comprehensive electrical output analysis. With optimized conditions, the W-TENG achieved a high open-circuit voltage of 58 V and a short-circuit current of 1.1 µA, and a power density of 59.5 mW/m^2^. To maximize the electrical output generated from the W-TENG, its structure was optimized by adjusting the spatial distance between the source and drain electrodes and the length of the source electrode. Furthermore, the correlation between hydraulic flow rates and electrical output was calibrated. Beyond energy harvesting, the W-TENG demonstrated exceptional sensitivity as a self-powered active sensor for real-time water-quality assessment. By leveraging the distinct surface charge densities and ion concentrations of different fluids, difference pH concentrations can be distinguished and diverse liquid compositions are classified based on the electrical outputs. This property of the W-TENG allows it to serve as a non-invasive monitoring sensor without the need for external power supplies or complex circuitry. To address practical power requirements, the synergistic effects of modular integration through series and parallel connections were investigated. The experimental results revealed a significant nonlinear enhancement in energy storage; specifically, the electrical energy stored in a 0.47 µF capacitor increased by 214% when utilizing optimized connections, far exceeding the sum of two single W-TENG units. As a proof of concept for environmental removal, the harvested energy was utilized to drive a self-powered electrochemical deposition system. By discharging the energy accumulated in a 1 mF capacitor, copper (Cu) ions, a representative heavy metal contaminant, were successfully removed from the aqueous environment. As a result, the proposed W-TENG offers an attractive and sustainable method for converting wasted hydraulic energy into electricity in pipe networks. Considering these results, the proposed W-TENG is expected to be utilized as a self-sustainable water purification and monitoring system in existing pipe infrastructures in the near future due to its significant potential as a self-sustainable water purification system.

## Figures and Tables

**Table 1:** The values of each parameter for evaluating the Cu deposition.

Parameter	Value	Parameter	Value
*q*_t_:	20.45 mg/g	*k*:	0.16 mg/min
*ECM*:	240.70 J/g	*E*_R_:	5.53%

## Data Availability

The data is contained within the article or Supplementary Materials.

## Supplementary Materials

The following supporting information can be downloaded at https://www.mdpi.com/article/10.3390/mi17020229/s1, Figure S1. The experimental configuration of the W-TENG. Figure S2. Transferred charge according to the water flow rate. Figure S3. Durability test of W-TENG. Figure S4. The change in the weight after electrochemical deposition. Table S1. A table showing the change in the weight after electrochemical deposition. Table S2. Definition of each parameter and formula.
