# Supplementary material for: A Triboelectricity-Driven Self-Sustainable System for Removing Heavy Metal from Water
Source: Micromachines (Basel). 2026 Feb 11;17(2):229. doi: 10.3390/mi17020229 (PMC12942936; doi:10.3390/mi17020229)
Supplement: Supplementary file 1 [file micromachines-17-00229-s001.zip › micromachines-4125637-supplementary.pdf]

Supplementary Materials

A Triboelectricity-Driven Self-Sustainable System for Removing Heavy Metal from Water

Jonghyeon Yun[1], Hyunwoo Cho[1], Geunchul Kim[2], Inkyum Kim[3] and Daewon Kim[2,3]*

1 Department of Electronics and Information Convergence Engineering, Kyung Hee University, 1732 Deogyeong-daero, Giheung-gu, Yongin 17104, Republic of Korea

2 Department of Semiconductor Engineering, Kyung Hee University, 1732 Deogyeong-daero, Giheung-gu, Yongin 17104, Republic of Korea

3 Department of Electronic Engineering, Institute for Wearable Convergence Electronics, Kyung Hee University, 1732 Deogyeong-daero, Giheung-gu, Yongin 17104, Republic of Korea

* Correspondence: daewon@khu.ac.kr;

Table of contents

1. **The experimental configuration of the W-TENG.**
2. **Transferred charge according to the water flow rate.**
3. **Durability test of W-TENG.**
4. **The change in the weight after electrochemical deposition**
5. **The table for change in the weight after electrochemical deposition**
6. **Definition of each parameter and formulas**

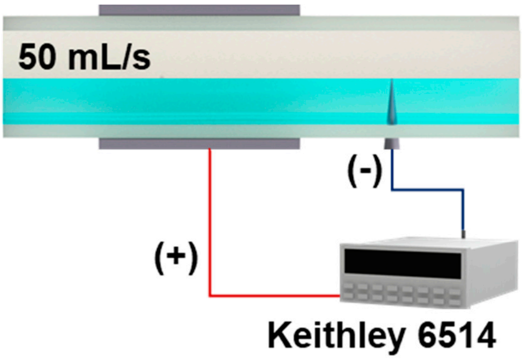

Figure S1. The experimental configuration of the W-TENG.

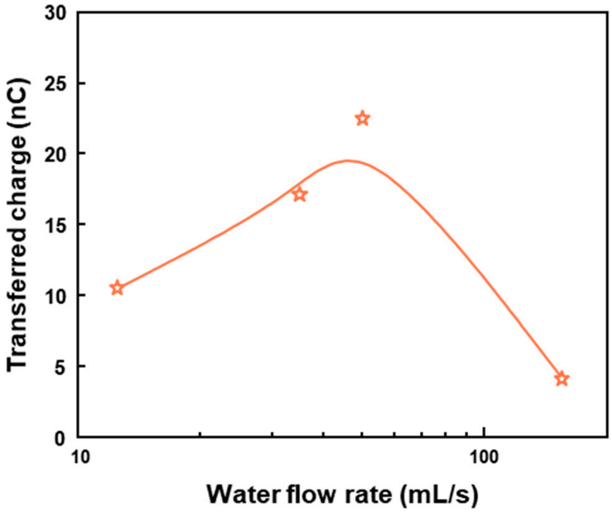

Figure S2. Transferred charge according to the water flow rate.

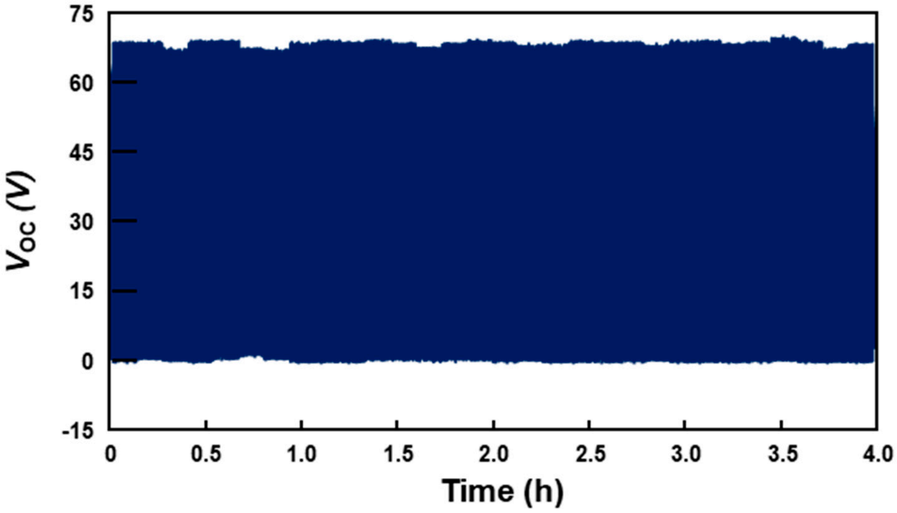

Figure S3. Durability test of W-TENG.

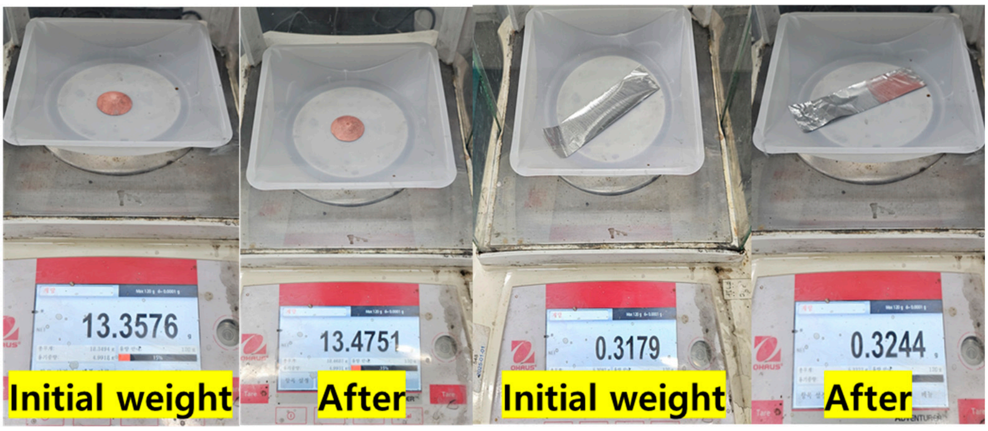

Figure S4. The change in the weight after electrochemical deposition (left: Cu source / right: Al electrode).

Initial weight	After deposition	ΔW (g)
Al: 0.3179 g	Al: 0.3244 g	ΔAl_W: 0.0065 g
Cu: 13.4751 g	Cu: 13.3576	ΔCu_W: 0.1175 g

Table S1. The change in the weight after electrochemical deposition.

Parameter	Formula
q_t	$\frac{(C_0 - C_t)V}{m}$,
	**C_0, C_t, V, and m are initial concentration of Cu, final concentration of Cu, volume, and mass of initial Al electrode.**
k	$\frac{dM_{Cu}}{dt}$
	**M_{Cu} and t are deposited weight of Cu on the Al electrode and time, and it is 0.16 mg/min.**
ECM	$\frac{\text{Total energy}}{dM_{\text{Cu}}}$

Table S2. Definition of each parameter and formulas.
